# Glucose values from the same continuous glucose monitoring sensor significantly differ among readers with different generations of algorithm

**DOI:** 10.1038/s41598-024-55124-3

**Published:** 2024-03-01

**Authors:** Naru Babaya, Shinsuke Noso, Yoshihisa Hiromine, Yasunori Taketomo, Fumimaru Niwano, Sawa Yoshida, Sara Yasutake, Yumiko Kawabata, Norikazu Maeda, Hiroshi Ikegami

**Affiliations:** Department of Endocrinology, Metabolism and Diabetes, Faculty of Medicine, Kindai University, 377-2 Ohno-higashi, Osaka-sayama, Osaka 589-8511 Japan

**Keywords:** Endocrine system and metabolic diseases, Diabetes

## Abstract

Continuous glucose monitoring (CGM) values obtained from CGM systems using the same sensor but with different internal algorithms (the first- and third-generation FreeStyle Libre (1st-gen-libre and 3rd-gen-libre, respectively)) were compared. We used 19,819 paired and simultaneously measured CGM values of 13 patients with diabetes. The average CGM value was significantly higher (*P* < 0.0001) and the time below range (CGM value < 70 mg/dL) was significantly lower (*P* < 0.0001) with the 3rd-gen-libre than with the 1st-gen-libre. There was a significant correlation (*P* < 0.0001) between the CGM values of the 3rd-gen-libre (y-axis, mg/dL) and 1st-gen-libre (x-axis, mg/dL) using the following formula: y = 0.9728x + 10.024. On assessing the association between glycated hemoglobin (HbA1c (%), y-axis) and the average CGM values (x-axis, mg/dL) by applying the obtained equation to previously reported 1st-gen-libre data and converting it to 3rd-gen-libre data, we obtained the equation y = 0.02628x + 3.233, indicating that the glucose management indicator reported in the West may be underestimated compared with the laboratory-measured HbA1c in the Japanese population. Glucose values from the same sensor were found to be significantly different between readers with different algorithms, and the calculation of CGM-related indices may need to be individualized for each device.

## Introduction

Intensive diabetes treatment has long-term beneficial effects, preventing the risk of microvascular and macrovascular complications^1^. To date, glycated hemoglobin (HbA1c) has long been the gold standard for assessing glycemic control and it is correlated with the risk of long-term complications^2^. Recently, new indicators derived from continuous glucose monitoring (CGM) devices, such as time above range (TAR), time in range (TIR), and time below range (TBR) have come into use in clinical practice and have established their position as more detailed short-term glycemic control indicators than HbA1c^2–4^. They are also effective in detecting and avoiding hypoglycemia, a complication that inevitably occurs when strict glycemic control is pursued^5,6^.

CGM devices consist of a sensor accompanied by a subcutaneous needle and a reader that reads and analyzes the information from the sensor. The principle of CGM is that the sensor measures the glucose level in the interstitial fluid and converts it into the equivalent venous blood glucose value using the algorithm in the reader. However, different CGM devices use different internal algorithms. Furthermore, even in the same CGM device, the internal algorithm changes depending on when it was launched. FreeStyle Libre (Abbott Japan Diabetes Care Inc., Tokyo, Japan) is currently available in Japan as an intermittently scanned CGM system. In 2021, the reader functionality was launched as a smartphone application. However, with this launch, the internal algorithm was changed from the first generation^7^ to the third generation^8^. Through daily clinical use of FreeStyle Libre for outpatients with diabetes, we noticed that the third-generation algorithm provides higher blood glucose levels and less frequent TBR values than the first-generation algorithm.

In this study, aiming to verify the clinical experience described above, we directly compared the CGM values derived from the first- and third-generation FreeStyle Libre (1st-gen-libre and 3rd-gen-libre, respectively) and examined the differences between them. Based on these actual data, we constructed a formula to make the 1st-gen-libre data accumulated so far compatible with the 3rd-gen-libre data that will be accumulated in the future. Using this newly constructed formula, the previously reported 1st-gen-libre data^9^ were converted to 3rd-gen-libre data, and the relationship between HbA1c and CGM-related indices was reexamined using the converted 3rd-gen-libre data.

## Results

**Figure 1:**
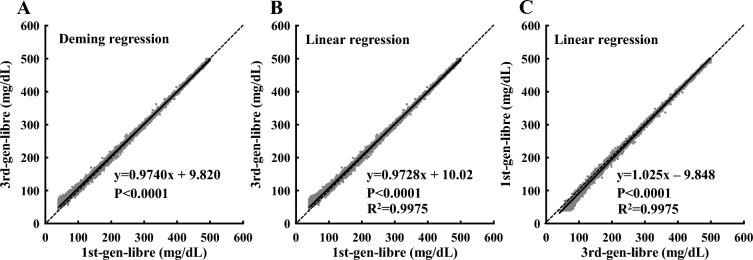
Scatterplot indicating the relationship between the CGM values from the 1st-gen-libre and 3rd-gen-libre. The dotted diagonal line is the line of identity, and the solid line is the line of best fit from Deming regression (**A**) and linear regression (**B**, **C**). Figures B and C show the x- and y-axes interchanged. 1st-gen-libre: first-generation FreeStyle Libre; 3rd-gen-libre: third-generation FreeStyle Libre, R^2^: coefficient of determination

A total of 19,819 pairs of 1st-gen-libre and 3rd-gen-libre CGM values were available. Figure 1 shows the results of the Deming regression and linear regression analysis. In the Deming regression (x-axis: 1st-gen-libre, y-axis: 3rd-gen-libre; Fig. 1A), the slope was 0.9740 (95% confidence interval [CI]: 0.9733–0.9747) and the y-intercept was + 9.820 mg/dL (95% CI 9.680–9.960), indicating a significant correlation between the two algorithms and that they are not identical, as the 95% CI for slope did not include 1 and the y-intercept did not include 0. To convert CGM values from the 1st-gen-libre to 3rd-gen-libre data and from the 3rd-gen-libre to 1st-gen-libre, linear regression analysis was performed, and the equations used for the estimation were y = 0.9728x + 10.02 and y = 1.025x − 9.848, respectively (Fig. 1B,C). There was a significant correlation between the CGM values of the 1st-gen-libre and 3rd-gen-libre, but when the CGM values were low (e.g., CGM value < 100 mg/dL), there was a high divergence between the CGM values.

**Figure 2:**
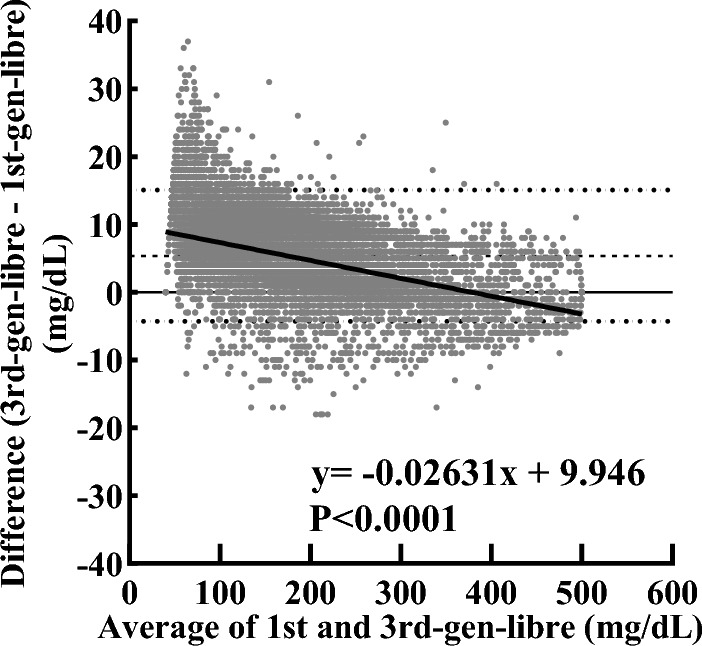
Bland–Altman plot of the difference in CGM value between the 3rd-gen-libre and 1st-gen-libre against the average CGM values of both. The long dashed horizontal line is the mean bias, and the two horizontal dotted lines represent the 95% confidence interval for agreement. The plot indicates that bias decreased linearly as the average value increased (*P* < 0.0001, linear regression). 1st-gen-libre: first-generation FreeStyle Libre; 3rd-gen-libre: third-generation FreeStyle Libre

To examine the error between 1st-gen-libre and 3rd-gen-libre due to differences in CGM values, Bland–Altman analysis was performed. The fixed bias was 5.385 mg/dL (95% CI − 4.288–15.06) (Fig. 2). In the linear regression, a slope of − 0.02631 (95% CI − 0.02701 to − 0.02561), a y-intercept of 9.946 mg/dL (95% CI 9.810–10.08), and an x-intercept of 378.1 mg/dL (95% CI 372.3–384.1) were shown, suggesting that the bias increased linearly as average glucose value decreased (*P* < 0.0001).

Table 1 shows the detailed analysis of CGM values for the 1st-gen-libre and 3rd-gen-libre. The mean CGM values were significantly higher with the 3rd-gen-libre than with the 1st-gen-libre (176.0 vs. 170.7 mg/dL, *P* < 0.0001, Mann–Whitney U test). The mean absolute difference was 5.9 mg/dL. When each data pair was compared between the 1st- and 3rd-gen-libre, there were 17,574 (out of 19,819; 88.7%) high values for the 3rd-gen-libre as compared with only 1324 (out of 19,819; 6.7%) for the 1st-gen-libre, and the number of equal CGM values was 921 (out of 19,819; 4.6%); the number of high CGM value was significantly larger for the 3rd-gen-libre (*P* < 0.0001, test for the proportion). When examining the CGM-related metrics, the distribution of TAR, TIR, and TBR was significantly different (*P* < 0.0001), with the frequency of TBR being significantly lower for the 3rd-gen-libre compared with the 1st-gen-libre (3.5 vs. 6.5%, *P* < 0.0001). Table 2 shows the detailed analysis of CGM values from the 1st-gen-libre and 3rd-gen-libre of each patient. The number of higher CGM values was significantly higher in all patients in the 3rd-gen-libre compared with the 1st-gen-libre (*P* < 0.0001, test for the proportion). The mean CGM values were also higher for all patients with the 3rd-gen-libre (significant differences were observed in 11 of 13 patients, except for 2 cases with higher CGM values). In the examination of CGM-related indices, TBR was lower for the 3rd-gen-libre than for the 1st-gen-libre in all patients except one, in whom hypoglycemia was not detected, suggesting that even when the same CGM device is used, the frequency of hypoglycemia detection decreases with different built-in algorithms.

**Table 1:** Summary of CGM values (n = 19,819) in 1st-gen-libre and 3rd-gen-libre.

	1st-gen-libre	3rd-gen-libre	P value
Mean CGM value ± SEM (mg/dL)	170.7 ± 0.6	176.0 ± 0.6	< 0.0001 ^b^
	(Mean absolute difference^a^ = 5.9 ± 0.0)		
Numbers of higher CGM values when each data pair was compared (19,819 pairs in total)	1,324 (6.7%)	17,574 (88.7%)	< 0.0001 ^c^
	(Number of equal CGM value: 921 (4.6%))		
CGM-related metrics			
TAR	36.4% (n = 7,223)	38.6% (n = 7,641)	< 0.0001 ^d^
TIR	57.1% (n = 11,312)	58.0% (n = 11,487)	
TBR	6.5% (n = 1,284)	3.5% (n = 691)	
TAR + TIR	93.5% (n = 18,535)	96.5% (n = 19,128)	< 0.0001 ^d^
TBR	6.5% (n = 1,284)	3.5% (n = 691)	

CGM, continuous glucose monitoring; n, number of paired data analyzed; TAR, time above range (CGM value ≥ 181 mg/dL); TBR, time below range (CGM value ≤ 69 mg/dL); TIR, time in range (CGM value 70–180 mg/dL); SEM, standard error of the mean; 1st-gen-libre and 3rd-gen-libre: the first- and third-generation FreeStyle libre.

^a^Absolute differences (AD) were calculated as follows: AD (mg/dL) =|1st-gen-libre–3rd-gen-libre|; ^b^Mann–Whitney U test; ^c^Test for the proportion if the null hypothesis is that the population proportion is 0.5; ^d^Chi-squared test.

**Table 2:** Relationship between CGM values obtained from 1st-gen-libre and 3rd-gen-libre in each patient.

Participant number	Age (years)	Sex	Number of paired data analyzed	Number of CGM values			Mean CGM values (mg/dL)			Difference (mg /dL)	MAD (mg /dL)	1st-gen-libre			3rd-gen-libre		
				Higher in 1st-gen-libre	Equal	Higher in 3rd-gen-libre	1st-gen-libre		3rd-gen-libre			TAR (%)	TIR (%)	TBR (%)	TAR (%)	TIR (%)	TBR (%)
1	45.3	M	1803	86	64	1653***	175.6		180.9*	5.3	5.6	43.3	52.6	4.1	46.3	51.9	1.8
2	33.2	M	1676	441	324	911***	338.5		339.9	1.4	3.3	92.4	7.6	0.0	93.1	6.9	0.0
3	24.1	F	1122	55	40	1027***	141.6		148.3**	6.7	7.2	31.6	44.3	24.2	32.9	50.1	17.0
4	61.0	F	3076	238	131	2707***	158.3		163.1***	4.9	5.5	33.6	61.6	4.8	35.7	62.0	2.3
5	41.4	F	617	178	62	377***	240.9		242.9	2.0	4.8	68.6	28.7	2.8	69.7	28.5	1.8
6	41.2	F	1342	29	13	1300***	118.4		127.1***	8.7	8.9	17.1	57.8	25.0	19.0	65.3	15.7
7	73.4	F	1894	46	76	1772***	158.1		163.1**	5.0	5.1	33.8	61.4	4.8	35.7	62.1	2.2
8	31.6	F	1706	89	69	1548***	165.6		170.6*	5.0	5.5	38.9	57.5	3.6	41.3	57.3	1.3
9	73.3	F	1016	48	13	955***	119.6		125.7***	6.1	6.4	10.6	75.8	13.6	12.1	82.1	5.8
10	55.9	M	1260	11	2	1247***	129.4		135.8***	6.3	6.4	6.8	91.9	1.3	8.6	91.3	0.2
11	48.2	F	1156	12	4	1140***	147.2		156.1***	8.9	9.0	22.8	75.2	2.1	26.3	73.3	0.4
12	32.6	F	951	86	64	1653***	134.5		139.8*	5.3	5.4	16.6	75.4	8.0	17.7	78.2	4.1
13	45.9	M	2200	441	324	911***	172.9		178.4**	5.5	5.6	42.5	56.0	1.5	45.9	53.9	0.2
Total	46.7 ± 4.3	F:9 M:4	19,819	1324	921	17,574***	170.7		176.0***	5.4	5.9	36.4	57.1	6.5	38.6	58.0	3.5

The number of higher CGM values in 3rd-gen-libre than in 1st-gen-libre was compared by a proportion test. Mean CGM values in 3rd-gen-libre and 1st-gen-libre were compared by Mann–Whitney U test. *P < 0.05, **P < 0.01, ***P < 0.001.

CGM, continuous glucose monitoring; F, female; M, male; MAD, mean absolute difference; TAR, time above range (CGM value ≥ 181 mg/dL); TBR, time below range (CGM value ≤ 69 mg/dL); TIR, time in range (CGM value 70–180 mg/dL); 1st-gen-libre and 3rd-gen-libre, the first- and third-generation FreeStyle libre.

**Figure 3:**
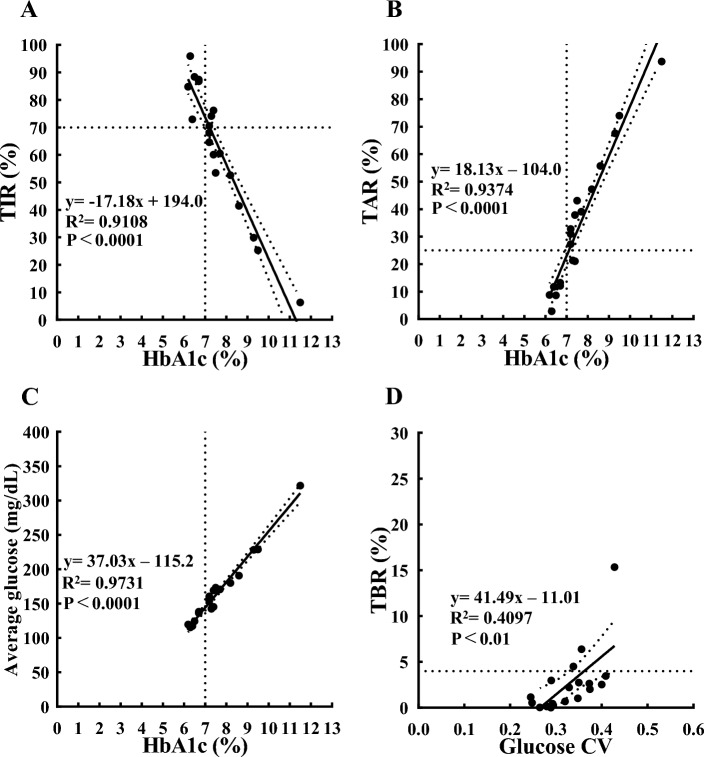
Scatterplot indicating the relationship between HbA1c (x-axis) and CGM-related metrics (TIR (**A**), TAR (**B**), and average glucose (**C**); y-axis), and between glucose CV (x-axis) and TBR (y-axis) (**D**). The solid line is the line of best-fit from the linear regression analysis. The dashed band is the 95% confidence band of the best-fit line. The dotted horizontal lines represent a TIR of 70% (**A**), a TAR of 25% (**B**), and a TBR of 4% (**D**) these values are based on the targets of TIR, TAR, and TBR for adults with type 1 or type 2 diabetes from recommendations of the ATTD 2019 consensus statement.^10^ The dotted vertical lines represent an HbA1c of 7% (**A**, **B**, and **C**). CV, coefficient of variation; TIR, time in range (glucose 70-180 mg/dL); TAR, time above range (glucose > 180 mg/dL); TBR, time below range (glucose < 70 mg/dL); R^2^, coefficient of determination

As mentioned above, the conversion formula from 1st-gen-libre to 3rd-gen-libre was y = 0.9728x + 10.02. Using this formula, we converted our previously reported dataset using 1st-gen-libre to 3rd-gen-libre data and reanalyzed it. In the analysis using the converted 3rd-gen-libre data to determine the relationship between HbA1c (x-axis) and TIR (y-axis), an HbA1c of 7% corresponded to a TIR of approximately 74%, and the coordinate (x = 7%, y = 70%) was included within the 95% confidence band of the best-fit line (Fig. 3A). For TAR, an HbA1c (x-axis) of 7% corresponded to a TAR (y-axis) of approximately 23%, and the coordinate (x = 7%, y = 25%) was included within the 95% confidence band of the best-fit line (Fig. 3B). The average CGM value was the index most strongly associated with HbA1c among the CGM-related indices (Fig. 3C). The equation for HbA1c (y-axis) deduced from the average CGM values (x-axis) was y = 0.02628x + 3.233 (Table 3). As reported in our previous study, the coefficient of variation of CGM values was significantly correlated with the TBR. The TBR corresponding to a CV of 0.36 was approximately 4% (Fig. 3D).

**Table 3:** Comparison of formulas for estimating HbA1c from average CGM values.

Report	Linear regression (y = eA1c or GMI (%), x = average CGM value (mg/dL))			Calculated HbA1c corresponding to a specific CGM average (%)			
	Estimating formula	95% CI of a slope	95% CI of a y-intercept when x = 0.0	100 mg/dL	140 mg/dL	180 mg/dL	220 mg/dL
eA1c *^a^	y = 0.02345x + 3.38	N/A	N/A	5.73	6.66	7.60	8.54
GMI *^b^	y = 0.02392x + 3.31	N/A	N/A	5.70	6.66	7.62	8.57
Previous our report*^c,^ *^d^	y = 0.02556x + 3.497	0.02339 – 0.02773	3.130 – 3.864	6.05	7.08	8.10	9.12
This report *^e^	y = 0.02628x + 3.233	0.02404 – 0.02852	2.844 – 3.622	5.86	6.91	7.96	9.01

CGM, continuous glucose monitoring; CI, confidence interval; eA1c: estimated HbA1c; GMI, glucose management indicator; NA, not available value.

*^a^Beck et al.^12^; *^b^Bergenstal et al.^13^; *^c^Babaya et al.^9^; *^d^Data using 1st-gen-libre; *^e^Data using the conversion formula from 1st-gen-libre to 3rd-gen-libre.

## Discussion

When comparing the CGM values derived from the same sensor in the CGM reading system of the same manufacturer but with different internal algorithms, significantly higher values were observed with the 3rd-gen-libre than with the 1st-gen-libre. Based on the data obtained, a formula was established to convert CGM values from the 1st-gen-libre to the 3rd-gen-libre data. Then, the established conversion formula was applied to previously published 1st-gen-libre data to examine the relationship between CGM-related indices and HbA1c after conversion to 3rd-gen-libre data, and an equation to convert the average CGM value into an HbA1c value was established.

First, we directly compared the 1st-gen-libre and 3rd-gen-libre data. The mean CGM values were significantly higher with 3rd-gen-libre than with the 1st-gen-libre (Fig. 2 and Table 1), and the difference in the CGM values was more pronounced for low CGM values (Fig. 2). In addition, the TBR was halved with the 3rd-gen-libre (Tables 1, 2), suggesting that an unexpectedly large number of hypoglycemia events have been detected in the past, and that there may have been a discrepancy between CGM-based glucose values and actual hypoglycemia based on actual glucose values and clinical symptoms or signs. These data indicate that we should be cautious about the fact that CGM devices from the same manufacturer may produce different results depending on the generation of the algorithm. Presumably, more differences may occur between instruments of different manufacturers. FreeStyle Libre Pro is available in Japan as a retrospective CGM device, but its internal algorithm is still the first generation one. Therefore, data obtained from FreeStyle Libre Pro and FreeStyle Libre, a personal CGM, should not be treated as identical.

Significant differences in CGM values derived from the 1st-gen-libre and 3rd-gen-libre prompted us to establish a conversion formula to convert the CGM values from 1st-gen-libre data to 3rd-gen-libre data. The conversion equation was y = 0.9728x + 10.02 (Fig. 1B). Using this equation, we re-analyzed the data collected in our previous study. In our previous paper examining the association between CGM-related indices and HbA1c, the CGM values were reported using the 1st-gen-libre, which was the only available device at the time. We examined the association between CGM-related indices and HbA1c after conversion to 3rd-gen-libre data. HbA1c of 7% corresponded to a TIR of 74% and TAR of 23% (previous study: 74% TIR and 20% TAR, respectively) (Fig. 3A,B). The 95% confidence interval of the best-fit line included the coordinates (HbA1c 7%, TIR 70%) and (HbA1c 7%, TAR 25%), suggesting that the criteria (TIR 70% and TAR 25%) based on data from the Western population, rather than the strict CGM index we have previously reported, would be sufficient to achieve an HbA1c of 7% in Japanese patients. In addition, there was a significant correlation between the CV of CGM values and TBR, and the CV corresponding to a TBR of 4% was 0.36 (Fig. 3D). Therefore, the TBR of 4% and CV of 0.36 used currently as control criteria^10,11^ were considered reasonable for the population in this study.

The CGM index that correlated best with the estimation of HbA1c from CGM values was the average CGM value. Indices such as eA1c^12^ and glucose management indicator (GMI)^13^ were based on the average CGM values, and the calculation formulas are summarized in Table 3. Currently, GMI, an improved version of eA1c, is widely used. In our clinical practice with Japanese patients with diabetes, we noticed a discrepancy between laboratory values of HbA1c and GMI (HbA1c > GMI). Therefore, we estimated the HbA1c using the average CGM values after correcting the CGM values obtained in the previous papers using the estimating equation developed in this study. When comparing the GMI and HbA1c obtained using the formula in this study, we found that the GMI was approximately 0.1–0.3% lower than the HbA1c value for average CGM values in the range of 100–220 mg/dL. Thus, the GMI may be somewhat underestimated compared with the laboratory-measured HbA1c value. In contrast, a report published in South Korea pointed out that the use of GMI may be overestimated^14^. One possible reason for this discrepancy could be the differences in CGM instrumentation. As shown in the present study, different generations of algorithms produced different average CGM values even with the same glucose sensor, suggesting that it is necessary to set GMI values for each device. Another reason may be the difference in background factors (race, diabetes type, obesity level, etc.). Differences in HbA1c and GMI have been reported to occur depending on race, type of diabetes, and body mass index^15,16^.

There are some limitations to this study; the first limitation is the temporal error in sensor readings. In order to minimize the error in CGM values as much as possible, we made an effort to use the 1st-gen-libre and the 3rd-gen-libre almost simultaneously, to recognize the sensor, and to match the built-in dates and times between them, but if the 3rd-gen-libre is started immediately after the 1st-gen-libre, there will be an error of a few seconds. During these few seconds, the readout frame of the libre can shift by as much as a minute, resulting in a slight error. Even when activated at the same time, there could be a slight difference in the time recorded for some reason. The differences, however, were mostly within 2 min; it is unlikely this was the reason for the higher CGM values obtained from the 3rd-gen-libre. A second limitation is that the comparative analysis of the 1st-gen-libre and 3rd-gen-libre data did not allow comparison with the gold standard–measured plasma glucose. Therefore, it is not known whether the measured plasma glucose levels are closer to those of the 1st-gen-libre or 3rd-gen-libre. A third limitation is that the calculation of CGM-related indices and the comparison of average CGM values with HbA1c were based on 3rd-gen-libre equivalent data rather than on direct 3rd-gen-libre data. Future correlation analysis studies with HbA1c using rigorous 3rd-gen-libre data, as we have conducted previously with the 1st-gen-libre^9^, are needed.

This study demonstrated that, even when the same sensor was used, there were significant differences in glucose values obtained from readers with different algorithms (1st-gen-libre and 3rd-gen-libre), with higher CGM values and less frequent detection of hypoglycemia with the 3rd-gen-libre than the 1st-gen-libre. The results also indicated that the GMI may be underestimated compared with the laboratory-measured HbA1c value, suggesting the need to individualize GMI values for each device. Our study sheds light on the differences between glucose monitoring devices.

## Methods

### Study design

This study was conducted at Kindai University Hospital. Among the outpatients with diabetes attending the hospital, 35 patients who used FreeStyle Libre were selected. Of these, 18 patients who were currently managing their diabetes using a data management page in the cloud (LibreLink) and using a smartphone (3rd-gen-libre) as a reader were included in this study. The patients’ data were collected in the usual daily life settings. The patients could start wearing the libre device whenever they wished. To ensure that the CGM values of the 1st-gen-libre and 3rd-gen-libre were directly comparable, several innovations were made. While the patients had smartphones, we also gave them a new reader (1st-gen-libre) that was adjusted to standard time. The patients were instructed to use the reader (1st-gen-libre) to activate the libre sensor, and immediately, it was recognized by an application on the smartphone. They were also asked to scan regularly with both the reader (1st-gen-libre) and smartphone (3rd-gen-libre). Data from the reader (1st-gen-libre), brought by the patient on the outpatient visit day, were uploaded to LibreLink and analyzed in combination with automatically uploaded data from the 3rd-gen-libre obtained from the smartphone application. The CGM values were categorized according to the target ranges of the glucose sensor, according to the international consensus report^10^: TAR (CGM, > 180 mg/dL), TIR (CGM, 70–180 mg/dL), and TBR (CGM, < 70 mg/dL). The average and coefficient of variation (CV) of the CGM values were also calculated. The protocols were approved by the Ethics Review Committee of the Kindai University Faculty of Medicine (approval number: 31–162), and all methods were performed in accordance with the relevant guidelines and regulations. Informed consent was obtained from the participants.

### Patients

Of the 18 patients described above, 13 who were able to comply with the study design were included in the analysis. Five patients were excluded due to missing CGM data (missing 1st-gen-libre data: four patients; missing 3rd-gen-libre data: one patient). The characteristics of the 13 patients are shown in Table 2. All the patients were adults (aged, 24–73 years). They received multiple-dose insulin injections or continuous subcutaneous insulin infusion for type 1 diabetes or pancreatectomy.

### Relationship of CGM-related indices with HbA1c: reanalysis of our previously reported data^9^

In the previous study, we calculated CGM-related indices using the 1st-gen-libre and examined their association with HbA1c^9^. In this study, we first derived a conversion equation from 1st-gen-libre to 3rd-gen-libre. Next, the 1st-gen-libre-based CGM values used in the previous study were converted to 3rd-gen-libre-equivalent CGM values. Furthermore, CGM-related indices were calculated from the 3rd-gen-libre equivalent CGM values, and their association with HbA1c was re-examined. The patient background and the study design are described in the previous paper^9^. Prior studies have examined the association between CGM-related indices and clinical measures during five periods of CGM value acquisition. In the current study, the CGM values from baseline to 120 days were used because they were the most accurate among the CGM values from different durations^9^.

### Units

In this study, we utilized mg/dL for CGM and % for HbA1c as the standard units. However, supplementary analyses were conducted using mmol/L for CGM and mmol/mol for HbA1c, and these results are provided in supplemental figures and supplemental tables. The conversion formulas applied were Glucose (mmol/L) = Glucose (mg/dL) × 0.05551 and HbA1c (mmol/mol) = 10.93 × HbA1c (%) - 23.52.

### Statistical analysis

Statistical analysis was performed by the Mann–Whitney U test for grouped data or Chi-squared test for categorical variables. The numbers of individuals with high CGM values on the 3rd-gen-libre and 1st-gen-libre were compared by a proportion test when the null hypothesis was set at 0.5. Deming regression was used to evaluate the relationship between 1st-gen-libre and 3rd-gen-libre values. Linear regression was used to estimate 3rd-gen-libre values from 1st-gen-libre and to estimate 1st-gen-libre values from 3rd-gen-libre. Bland–Altman plot analysis was performed to compare the bias between 1st-gen-libre and 3rd-gen-libre values. Statistical analysis was performed using GraphPad Prism®, version 9.5.1, statistical software (San Diego, California). *P* < 0.05 was considered statistically significant.

### Supplementary Information

Supplementary Information.

## Data Availability

The datasets used and/or analyzed during the current study available from the corresponding author on reasonable request.
