# Supplementary material for: Glucose values from the same continuous glucose monitoring sensor significantly differ among readers with different generations of algorithm
Source: Sci Rep. 2024 Mar 1;14:5099. doi: 10.1038/s41598-024-55124-3 (PMC10907350; doi:10.1038/s41598-024-55124-3)
Supplement: Supplementary file 1 — Supplementary Information. [file 41598_2024_55124_MOESM1_ESM.pdf]

Glucose values from the same continuous glucose monitoring sensor significantly differ among readers with different generations of algorithm

Naru Babaya, Shinsuke Noso, Yoshihisa Hiromine, Yasunori Taketomo, Fumimaru Niwano, Sawa Yoshida, Sara Yasutake, Yumiko Kawabata, Norikazu Maeda, and Hiroshi Ikegami

Department of Endocrinology, Metabolism and Diabetes, Kindai University Faculty of Medicine, 377-2 Ohno-higashi, Osaka-sayama, Osaka 589-8511, Japan

Supplementary Figure S1

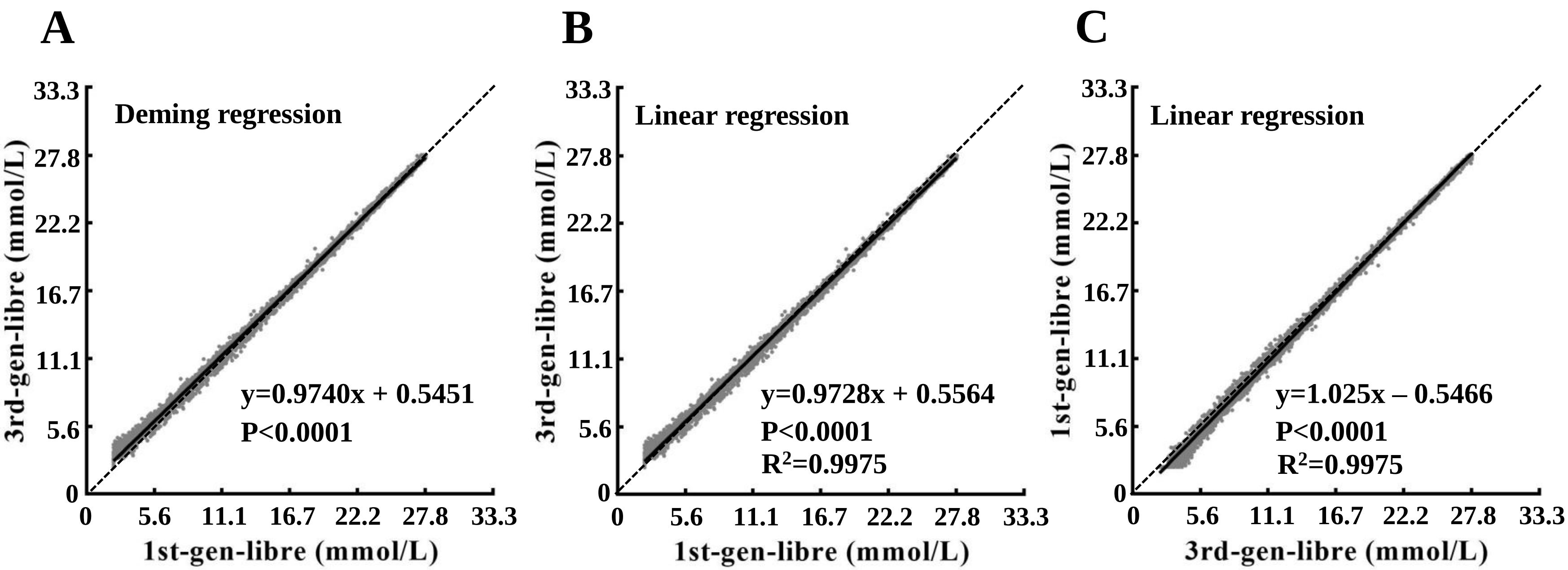

Supplementary Figure S2

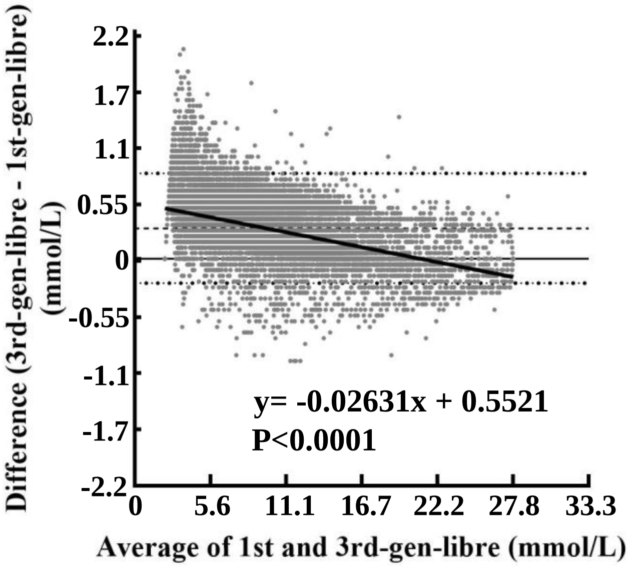

Supplementary Figure S3

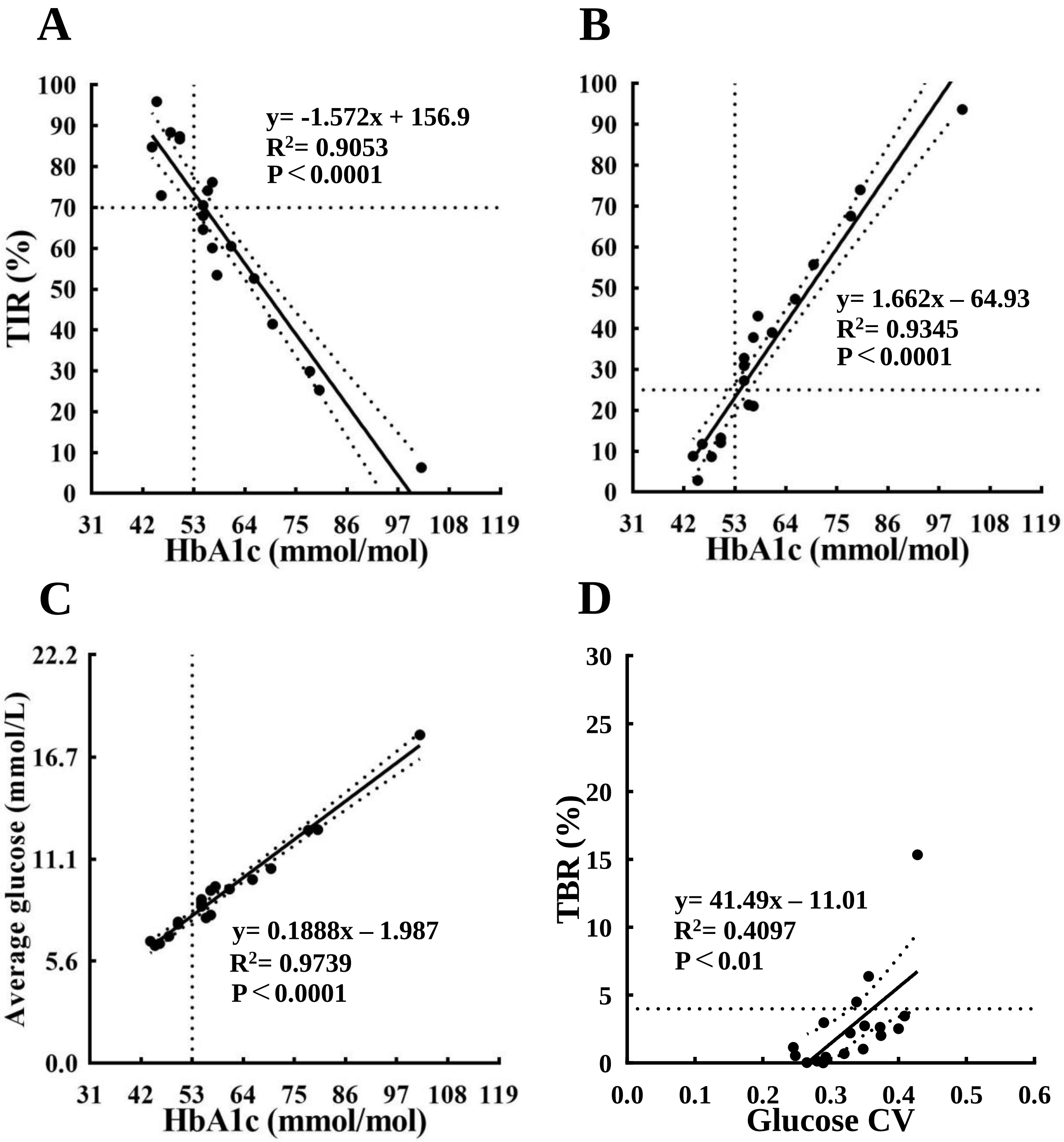

Supplementary Table S1. Summary of CGM values (n=19,819) in 1st-gen-libre and 3rd-gen-libre

	1st-gen-libre	3rd-gen-libre	P value
Mean CGM value ± SEM (mmol/L)	9.47 ± 0.03	9.77 ± 0.03	< 0.0001 [b]
	(Mean absolute difference [a] = 0.33 ± 0.0)		
Numbers of higher CGM values when each data pair was compared (19,819 pairs in total)	1,324 (6.7%)	17,574 (88.7%)	< 0.0001 [c]
	(Number of equal CGM value: 921 (4.6%))		
CGM-related metrics			
TAR	36.4% (n=7,223)	38.6% (n=7,641)	< 0.0001 [d]
TIR	57.1% (n=11,312)	58.0% (n=11,487)	
TBR	6.5% (n=1,284)	3.5% (n=691)	
TAR + TIR	93.5% (n=18,535)	96.5% (n=19,128)	< 0.0001 [d]
TBR	6.5% (n=1,284)	3.5% (n=691)	

[a] Absolute differences (AD) were calculated as follows: AD (mmol/L) = |1st-gen-libre - 3rd-gen-libre|. [b] Mann–Whitney U test. [c] Test for the proportion if the null hypothesis is that the population proportion is 0.5. [d] Chi-squared test.

CGM: continuous glucose monitoring; n=Number of paired data analyzed; TAR: time above range (CGM value ≥10.0 mmol/L); TBR: time below range (CGM value ≤3.8 mmol/L); TIR: time in range (CGM value 3.9-9.9 mg/dL); SEM: standard error of the mean; 1st-gen-libre and 3rd-gen-libre: the first- and third-generation FreeStyle libre

Supplementary Table S2. Relationship between CGM values obtained from 1st-gen-libre and 3rd-gen-libre in each patient

Participant number	Age (years)	Sex	Number of paired data analyzed	Number of CGM values			Mean CGM values (mmol/L)		Difference (mmol/L)	MAD (mmol/L)	1st-gen-libre			3rd-gen-libre		
				Higher in 1st-gen-libre	Equal	Higher in 3rd-gen-libre	1st-gen-libre	3rd-gen-libre			TAR (%)	TIR (%)	TBR (%)	TAR (%)	TIR (%)	TBR (%)
1	45.3	M	1803	86	64	1653***	9.75	10.04*	0.29	0.31	43.3	52.6	4.1	46.3	51.9	1.8
2	33.2	M	1676	441	324	911***	18.65	18.73	0.08	0.18	92.4	7.6	0.0	93.1	6.9	0.0
3	24.1	F	1122	55	40	1027***	7.86	8.23**	0.37	0.40	31.6	44.3	24.2	32.9	50.1	17.0
4	61.0	F	3076	238	131	2707***	8.79	9.06***	0.27	0.31	33.6	61.6	4.8	35.7	62.0	2.3
5	41.4	F	617	178	62	377***	13.37	13.48	0.11	0.27	68.6	28.7	2.8	69.7	28.5	1.8
6	41.2	F	1342	29	13	1300***	6.57	7.05***	0.48	0.49	17.1	57.8	25.0	19.0	65.3	15.7
7	73.4	F	1894	46	76	1772***	8.77	9.05**	0.28	0.28	33.8	61.4	4.8	35.7	62.1	2.2
8	31.6	F	1706	89	69	1548***	9.19	9.47*	0.28	0.30	38.9	57.5	3.6	41.3	57.3	1.3
9	73.3	F	1016	48	13	955***	6.64	6.98***	0.34	0.36	10.6	75.8	13.6	12.1	82.1	5.8
10	55.9	M	1260	11	2	1247***	7.19	7.54***	0.35	0.36	6.8	91.9	1.3	8.6	91.3	0.2
11	48.2	F	1156	12	4	1140***	8.17	8.67***	0.49	0.50	22.8	75.2	2.1	26.3	73.3	0.4
12	32.6	F	951	86	64	1653***	7.47	7.76*	0.29	0.30	16.6	75.4	8.0	17.7	78.2	4.1
13	45.9	M	2200	441	324	911***	9.60	9.90**	0.30	0.31	42.5	56.0	1.5	45.9	53.9	0.2
Total	46.7 ±4.3	F:9 M:4	19819	1324	921	17574***	9.47	9.77***	0.30	0.33	36.4	57.1	6.5	38.6	58.0	3.5

The number of higher CGM values in 3rd-gen-libre than in 1st-gen-libre was compared by a proportion test. Mean CGM values in 3rd-gen-libre and 1st-gen-libre were compared by Mann–Whitney U test. *P<0.05, **P<0.01, ***P<0.001.

CGM: continuous glucose monitoring; F: female; M: male; MAD: Mean absolute difference; TAR: time above range (CGM value ≥10.0 mmol/L); TBR: time below range (CGM value ≤3.8 mmol/L); TIR: time in range (CGM value 3.9-9.9 mmol/L); 1st-gen-libre and 3rd-gen-libre: the first- and third-generation FreeStyle libre.

Supplementary Table S3. Comparison of formulas for estimating HbA1c from average CGM values

Report	Linear regression (y=eA1c or GMI (mmol/mol), x= average CGM value (mmol/L))			Calculated HbA1c corresponding to a specific CGM average (mmol/mol)			
	Estimating formula	95% CI of a slope	95% CI of a y-intercept when x=0.0	5.6 mmol/L	7.8 mmol/L	10.0 mmol/L	12.2 mmol/L
eA1c [*1]	N/A	N/A	N/A	N/A	N/A	N/A	N/A
GMI [*2]	y=4.70587x + 12.71	N/A	N/A	39.1	49.4	59.8	70.1
Previous our report [*3, *4]	y=5.019x + 14.68	4.599 – 5.438	10.75 – 18.62	42.8	53.8	64.9	75.9
This report [*5]	y=5.160x + 11.81	4.728 – 5.592	7.641 – 15.98	40.7	52.1	63.4	74.8

[*1] Beck, RW. et al. Diabetes Care 40, 994 (2017). [*2] Bergenstal, RM. et al. Diabetes Care 41, 2275 (2018). [*3] Babaya, N. et al. Sci Rep 11, 4006 (2021). [*4] Data using 1st-gen-libre. [*5] Data using the conversion formula from 1st-gen-libre to 3rd-gen-libre.

CGM: continuous glucose monitoring; CI: confidence interval; eA1c: estimated HbA1c; GMI: glucose management indicator; NA: Not available value.
